# Prenatal exposure to aromatic amines and associations with infant cognition in the ECHO.CA.IL cohorts

**DOI:** 10.1038/s41370-026-00905-3

**Published:** 2026-09-21

**Authors:** Stephanie M. Eick, Kaegan Ortlund, Amy M. Padula, Kurunthachalam Kannan, Andréa Aguiar, Joshua F. Robinson, Rachel Morello-Frosch, Susan L. Schantz, Tracey J. Woodruff

**Affiliations:** 1 Gangarosa Department of Environmental Health, Rollins School of Public Health, Emory University, Atlanta, GA USA; 2 Program on Reproductive Health and the Environment, Department of Obstetrics, Gynecology and Reproductive Sciences, University of California, San Francisco, San Francisco, CA USA; 3 Wadsworth Center, New York State Department of Health, Empire State Plaza, Albany, NY USA; 4 Department of Environmental Health Sciences, State University of New York at Albany, Albany, NY USA; 5 Beckman Institute for Advanced Science and Technology, University of Illinois Urbana-Champaign, Champaign, IL USA; 6 Department of Comparative Biosciences, University of Illinois Urbana-Champaign, Champaign, IL USA; 7 Center for Reproductive Sciences, Department of Obstetrics, Gynecology and Reproductive Sciences, University of California, San Francisco, San Francisco, CA USA; 8 Department of Environmental Science, Policy and Management and School of Public Health, University of California, Berkeley, Berkeley, CA USA

**Keywords:** Aromatic amines, Neurodevelopment, exposure assessment, Mixture

## Abstract

**Background:**

Aromatic amines (AAs) are carcinogenic compounds widely used in consumer products. However, few studies have assessed exposure to AAs during pregnancy, and no human studies have examined associations between AAs and infant cognition, despite in vitro evidence of neurotoxicity.

**Objective:**

We conducted an analysis of prenatal AA exposure and infant cognitive outcomes using a subset of the Illinois Kids Development Study and Chemicals in Our Bodies, two prospective birth cohorts that together make up ECHO.CA.IL (*N* = 250).

**Methods:**

AAs, including aniline, *o*/*m*-toluidine, 4-chloroaniline, *o*-anisidine, 4,4’methylenedianiline, and cotinine, were measured in 2^nd^ trimester urine samples and detected in >50% of participants. Infant cognition was assessed using a visual recognition memory (VRM) task administered using an infrared eye tracker at 7.5 months. We used linear regression to examine associations between individual natural log transformed AAs and their molar sum with VRM outcomes. We examined effect modification and interaction by child sex.

**Results:**

A natural log unit increase in ΣAAs and the individual analyte 4,4’methylenedianiline was associated with an increase in time to reach familiarization, suggesting poorer attention (β = 7.1, 95% confidence interval [CI] = -0.97, 15.16; β = 3.95, 95% CI = -0.27, 8.17, respectively). Cotinine was associated with worsening novelty preference (β=-0.01, 95% CI = -0.02, 0), indicating poorer working memory. Aniline was associated with an increase in time to reach familiarization only among females (*p*-value for interaction=0.06).

**Impact:**

- This is the first epidemiologic study to evaluate prenatal exposure to aromatic amines in relation to infant cognition. We leverage biomarkers of aromatic amines exposure during pregnancy and a sensitive, eye-tracking–based visual recognition memory task at 7.5 months to capture early cognitive domains rarely assessed in environmental epidemiology. By examining individual compounds, their molar sum, and sex-specific effects within a prospective cohort, this study extends concerns about aromatic amines beyond carcinogenicity to early neurodevelopment, highlighting a previously unrecognized pathway of potential harm during a critical developmental window.

## Introduction

Aromatic amines (AAs) are a class of environmental chemicals characterized by an amine group directly attached to a benzene ring [1]. They are widely used in the production of dyes and pigments and are also present in rubber, plastics, explosives, pesticides, and certain pharmaceuticals. Additional exposures occur through tobacco smoke, diesel exhaust, and high-temperature cooking of meats [2].

Although, AA exposures are widespread, they are not routinely monitored in human population-based studies, including among pregnant women [3]. In the ECHO cohort, several AAs (*n* = 9) were reported to be commonly detected ( > 60%) in maternal urine, with higher levels of 3,4-dichloroaniline, 4,4’-methylenedianiline, and 4-chloroaniline observed in certain subgroups [3]. Specific AAs were also positively associated with cotinine, the primary metabolite of nicotine and a biomarker of tobacco smoke exposure, suggesting common co-exposures [3].

While many AAs are recognized toxicants, their developmental health risks remain poorly defined. The International Agency for Research on Cancer (IARC) has classified several AAs, including aniline, *o*-toluidine, 4-chloroaniline, *o*-anisidine, and 4,4’methylenedianiline, as probable or possible human carcinogens [4]. and many are also listed under California’s Proposition 65 as chemicals known to cause cancer or reproductive harm [5]. Despite these classifications, few studies have evaluated the potential impacts of prenatal AA exposure, even though these compounds readily cross the placental barrier [6].

Aromatic amines were identified by the ECHO program as a priority chemical based on evidence of exposure and potential for neurodevelopmental harm [7]. Experimental evidence supports concern for developmental neurotoxicity from several AAs. In mice, prenatal aniline exposure reduced neuron number in the sexually dimorphic nucleus of the preoptic area, an effect linked to altered neurobehavioral outcomes [8]. In non-mammalian models, halogenated anilines produced malformations in *Xenopus* embryo, including spinal defects [9] while 3,4-dichloroaniline induced edema, spinal deformities, mortality and impaired locomotor activity in zebrafish [10]. In rats, gestational exposure to 3,5-dimethylaniline caused cortical thinning and reduced cortical cell counts [11]. Animal studies further find that acute exposures to aniline and halogenated anilines led to motor deficits, gait abnormalities, reduced grip strength, and neuropathological changes in the spinal cord and brainstem [12, 13].

Mechanistic studies indicate that anilines and other common AAs induce DNA damage, oxidative stress, and inflammation and/or inhibit cell proliferation [11, 14–18]. pathways relevant to neurocellular injury, cognitive impairment, developmental delay, and pregnancy complications such as preterm birth [19, 20]. In humans, a Brazilian pregnancy cohort (*n* = 300), reported widespread detection of urinary AAs (100% detection rate for eight analytes) [21]. Specific compounds (e.g., 2-napthylamine and 4-aminobiphenyl) were positively correlated with 8-hydroxy-2’-deoxyguanosine (8-OH-dG), a well-established biomarker of oxidative stress and DNA damage that has been linked to neurodevelopmental deficits [21, 22].

Cotinine itself has been linked to neurodevelopmental deficits [23]. though interpretation is complicated by differences in toxicity between nicotine, cotinine, and tobacco smoke mixtures. Nonetheless, rodent studies demonstrate prenatal nicotine and cotinine exposures lead to long-lasting deficits in spatial memory and attention, along with structural changes in brain regions critical for learning and memory [24].

These studies identify the importance of further studies to evaluate whether prenatal exposures influence early cognition, a timepoint when there may be opportunities to intervene more effectively and improve longer-term cognitive outcomes. However, this has not been studied to date in humans. In the present study, we pooled data from two longitudinal birth cohorts in San Francisco, California and Urbana-Champaign, Illinois. We assessed whether prenatal exposures to AAs, individually and as a mixture, were associated with infant cognition at 7.5 months, hypothesizing that elevated levels of AAs would be associated with poorer cognitive outcomes. Notably, we assessed infant cognition using a novel visual recognition memory (VRM) task, which uses infrared eye-tracking to obtain measures of cognitive functions including visual attention, information processing speed and working memory that are considered to be the basic building blocks of cognition [25]. Importantly, the outcome measures assessed in this study have been previously found to be predictive of cognitive function at later developmental stages using a non-automated VRM task [26–28].

## Methods

### Study population

Participants included in this analysis were a subset of mother-child pairs enrolled in the Illinois Kids Development Study (IKIDS) and Chemicals in Our Bodies (CIOB) cohort. These prospective birth cohorts were merged between 2016 and 2023 to form the ECHO.CA.IL prospective cohort. The subset of participants included in this analysis were assessed on the VRM task available at 7.5 months and had a prenatal urine sample analyzed for AAs (*N* = 250 total, 186 in IKIDS and 64 in CIOB). Detailed information regarding recruitment, enrollment, and eligibility criteria for these two cohorts is provided elsewhere [29]. Briefly, participants in IKIDS were recruited from clinics in Urbana, IL. Eligibility criteria were: singleton pregnancy at less than 15 weeks gestation, between 18 and 40 years of age at the start of pregnancy, spoke English as a primary language, no known pregnancy complications, not already enrolled in the study with another child, resided within a 30-min drive of the University of Illinois in Urbana-Champaign, IL and not planning to move out of the study area before the child’s first birthday. Participants in CIOB were recruited from University of California, San Francisco (UCSF) hospitals in the San Francisco Bay Area of California. Pregnant women were eligible for inclusion if they were at least 18 years of age at enrollment, not pregnant with multiples, in the second trimester, and English or Spanish-speaking.

Upon enrollment, participants in both cohorts completed a self-reported interview questionnaire that was used to ascertain information regarding maternal age, maternal education, marital status, and maternal race/ethnicity. Information regarding parity and child sex, and pre-pregnancy body mass index (BMI; kg/m^2^) were obtained via medical record abstraction in CIOB and maternal self-report in IKIDS.

### Ethics approval and consent to participate

The IKIDS protocol was approved by the Institutional Review Board (IRB) at the University of Illinois at Urbana Champaign and the CIOB protocol was approved by the UCSF and University of California, Berkeley IRBs (IRB approval #s 09498, 10-0086, and 2010-05-04, respectively*)*. All participants provided written, informed consent prior to participating. All methods were performed in accordance with the relevant guidelines and regulations.

### Measurement of aromatic amines (AAs)

Urine samples were obtained from CIOB and IKIDS participants during the second trimester. In both cohorts, urine samples were frozen at -80˚C prior to analysis of AAs, which was conducted at the Wadsworth Center Human Health Exposure Analysis Resource (HHEAR) laboratory. Samples were analyzed for AAs using high-performance liquid chromatography-tandem mass spectrometry using validated methods that have previously been described in detail [3, 30]. Quantification was based on isotope dilution using labeled internal standards. We focused on AAs that were detected in at least 50% of urine samples, which included: aniline, cotinine, *o*/*m*-toluidine, 4-chloroaniline, *o*-anisidine, and 4,4’methylenedianiline (all in ng/mL). Coefficients of variations (CVs) obtained from twenty internal lab matrix spikes or analytes included in this analysis were low, ranging from 6 to 30%. In addition to examining all analytes individually, we calculated the molar sum of AAs (in μM) using the equation [31].

$$\Sigma {{\rm{AA}}}= 	\frac{{{\rm{aniline}}}}{93.13}+\frac{{{\rm{cotinine}}}}{176.21}+\,\frac{o/m-{{\rm{toluidine}}}}{107.15}+\frac{\,4\,{{\rm{chloroaniline}}}}{127.57}\\ 	 +\frac{o-{{\rm{anisidine}}}}{123.15}+\frac{{{\mathrm{4,4}}}^{{\prime} }{{\rm{methylenedianiline}}}}{198.26}\,$$

Values below the limit of detection (LOD) were imputed with LOD divided by the square root of 2. All individual analytes, as well as the sum, were right skewed and natural log transformed prior to statistical analysis. To account for urinary dilution, we included specific gravity as a covariate in all regression models [32]. We also included cotinine as a binary variable (detect versus non-detect) in regression models given that approximately 55% of participants had a detectable value.

### Visual recognition memory

To complete the task, infants sat on a parent’s lap in a dark booth surrounded by curtains and in front of a large screen HD TV screen. During familiarization trials, infants were shown two identical black and white photographs of human faces (the familiarization stimuli) side-by-side on the television screen. Once the infant accumulated a total of 20 s looking at the identical pair of faces, they were given two test trials where the infant was shown the now familiar face paired with a novel face (the test stimulus). In IKIDS, all test trials were counterbalanced so that half of the infants saw stimuli set A as the familiar stimulus and set B as the novel stimulus (Set 1), and the other half of the infant sample saw the reverse: set B was the familiar stimulus and set A was the novel (Set 2). Additionally, within Set 1 and Set 2, half of the infant sample saw the novel stimulus on the right side in the first test trial, and on the left side in the second test trial, and the other half of the sample saw the novel stimulus on the left side first, and then on the right side. Counterbalancing for these two variables is important to address infants’ spontaneous preferences for looking at a particular stimulus over another and looking longer at objects appearing to the right of their field of vision. Because infants’ inherent visual attention biases were already addressed in the IKIDS sample, and because CIOB had a smaller infant sample, all CIOB infants were tested using Set 2 and with the novel stimulus appearing first on the left and then on the right. The complete VRM task consisted of five blocks of 1 familiarization and 2 test trials, with a different pair of familiar and novel stimuli displayed in each block. For this analysis, we utilized data from the first two blocks of trials only, as our prior work in IKIDS indicated that these initial two blocks are the most sensitive [25]. Infants’ responses during the two test trials were averaged for analysis to counteract infants’ right-side attentional bias.

The VRM assessment used in this study was a paired-comparison VRM paradigm based on previous literature finding that infant cognition at 7 months is predictive of cognitive abilities at 6 years of age [33]. A detailed characterization of the VRM task in the IKIDS sample has been provided elsewhere [25]. Eye tracking data were extracted and processed using DataViewer software (SR Research Lt, Mississauga, Ontario, Canada) as previously described [25]. Using DataViewer, we defined interest areas for each trial and stimulus and extract information regarding trial durations, visual fixations, blinks, and saccades within, between, and outside interest areas. Using the data from the two initial blocks of trials, we calculated four indicators of cognition. Time to reach familiarization (i.e., how long infants took to accumulate 20 s of looking at the familiarization stimulus in each block) was used to assess infants’ visual attention, as a longer period being “off-task” or looking away from the target stimulus suggests poorer visual attention. Average run duration (i.e., the average time an infant spent looking at the two identical familiarization stimuli before looking away) was included as a measure of information processing speed, with shorter run durations or periods of time examining the stimulus indicating faster, more efficient, and thus, better information processing. A third indicator obtained from the familiarization trial was shift rate (i.e., the rate at which the infants shifted their gaze between the two stimuli) was included as an additional measure of visual attention, with higher shift rates indicating that the infant was making more comparisons between the two stimuli and thus more actively engaged in the task. The fourth and last indicator, which came from the averaged test trials, was novelty preference (i.e., the proportion of time the infant looked at the novel face as compared to the familiar face) and measured working memory. Infants have a preference for novelty, thus a higher proportion of time looking at the novel face indicates better memory for the stimulus displayed in the familiarization trials [34].

### Statistical analysis

We examined the distribution of maternal and infant characteristics in the pooled ECHO.CA.IL cohort and individual cohorts using frequencies and counts for categorical variables and means and standard deviations (SDs) for continuous variables. To examine distributions of AAs in the pooled and individual cohorts, we calculated geometric means (GMs), geometric SDs (GSDs), and selected percentile distributions. Spearman correlation coefficients were calculated to assess correlations between natural log transformed AAs in the pooled cohort.

Adjusted and minimally adjusted generalized linear regression models were used to examine associations between individual natural log transformed AA concentrations and their sum in relation to each of the four VRM outcomes. In pooled models, we adjusted for cohort (CIOB, IKIDS), urinary specific gravity (continuous), maternal age (continuous), maternal education ( < college degree, college degree, graduate degree), pre-pregnancy BMI (continuous), child age at VRM assessment (continuous, corrected for prematurity, defined as child age in weeks minus weeks preterm), and child sex (male, female). Child sex, urinary specific gravity, and child age at VRM assessment were included as precision variables in adjusted models. Minimally adjusted models included urinary specific gravity. Covariates retained in adjusted models were identified via a literature review that was used to inform a directed acyclic graph (DAG). We additionally examined associations with exposures and outcomes in our study population. We did not adjust for smoking status as very few participants in our analytic sample reported smoking during pregnancy. CIOB-specific models adjusted for maternal education as a binary variable ( ≤ college degree, graduate degree) due to positivity concerns. IKIDS specific models were additionally adjusted for stimulus set.

To further examine univariate associations and interactions between exposures, we used Bayesian kernel machine regression (BKMR) [35, 36]. BKMR models were run using 10,000 iterations and component wide variable selection. Next, we estimated univariate exposure response functions to assess associations between individual AAs and each VRM outcome while holding the AAs constant at their 50^th^ percentile. To assess interactions between exposures, we calculated single variable risk summaries, which estimated the change in each VRM outcome associated with increasing a single exposure from the 25^th^ to 75^th^ percentile while holding other exposures constant at specific percentiles.

Lastly, we conducted several sensitivity analyses. We stratified linear regression models by child sex, as prior work indicates that effects of AAs are sex specific and our prior work has observed sex specific associations between various chemical exposures on this VRM task [31, 37]. We calculated *p*-values for interaction (AA*sex) in models that were not stratified. Within IKIDS, we stratified by set and in unstratified models calculated *p*-values for interaction (AA*set). *P*-values < 0.05 and *p* values for interaction <0.1 were considered statistically significant. All analyses were conducted in R version 4.5.0 and models were run on complete cases (*N* = 233).

## Results

There were 250 participants included in our ECHO.CA.IL analytic sample, of which 186 were enrolled in IKIDS and 64 were enrolled in CIOB. Within the pooled ECHO.CA.IL cohort, the mean maternal age was 32 years (SD = 4.2), and the mean pre-pregnancy BMI was 26 kg/m^2^ (SD = 6.4) (Table 1). Most participants were married or living with a partner (93%), had obtained a graduate degree (50%) and were multi-parous (55%). More participants in IKIDS identified as White compared to CIOB (83% versus 47%), while slightly more participants in CIOB delivered a female infant (59% versus 53%). The distributions of demographic characteristics in the larger cohorts is similar to those included in our analytic sample (Table S1). When examining the distribution of VRM outcomes, we observed that novelty preference was slightly higher in IKIDS relative to CIOB (0.6 versus 0.5), which is likely the result of half of infants in IKIDS seeing set 1, where the preferred stimulus was the novel one. The mean time to reach familiarization was shorter in IKIDS relative to CIOB (46.7 versus 59.7) (Table 1).

**Table 1:** Distribution of demographic characteristics & visual recognition memory (VRM) outcomes in a subset of the Chemical in Our Bodies cohort (CIOB), Illinois Kids Developmental Study cohort (IKIDS), and pooled ECHO.CA.IL cohort.

	CIOB	IKIDS	ECHO.CA.IL
	*N* = 64	*N* = 186	*N* = 250
	*N* (%) or Mean (SD)	*N* (%) or Mean (SD)	*N* (%) or Mean (SD)
Demographics			
Maternal Age (years)	33.61 (4.79)	30.92 (3.73)	31.61 (4.19)
Pre-pregnancy body mass index (kg/m^2^)	24.10 (4.57)	26.58 (6.70)	26.05 (6.38)
Missing	14	1	15
Marital Status			
Married/Living with a partner	59 (94%)	173 (93%)	232 (93%)
Single	4 (6.3%)	13 (7.0%)	17 (6.8%)
Missing	1		1
Maternal Education			
<College degree	13 (20%)	26 (14%)	39 (16%)
College degree	20 (31%)	66 (35%)	86 (34%)
Graduate degree	31 (48%)	94 (51%)	125 (50%)
Maternal Race/Ethnicity			
Asian/Native Hawaiian/Pacific Islander/American Indian/Alaska Native	17 (27%)	10 (5.4%)	27 (11%)
Black/Multi-racial	10 (16%)	17 (9.1%)	27 (11%)
Hispanic	7 (11%)	5 (2.7%)	12 (4.8%)
White	30 (47%)	154 (83%)	184 (74%)
Parity			
No prior births	31 (49%)	80 (43%)	111 (45%)
1 or more prior births	32 (51%)	106 (57%)	138 (55%)
Missing	1		1
Child Sex			
Male	26 (41%)	88 (47%)	114 (46%)
Female	37 (59%)	98 (53%)	135 (54%)
Missing	1		1
Child Age (days)	243.10 (10.06)	245.34 (17.82)	244.77 (16.22)
Missing	1	1	2
VRM			
Shift Rate	0.39 (0.18)	0.46 (0.20)	0.44 (0.19)
Novelty Preference (%)	0.53 (0.10)	0.57 (0.10)	0.56 (0.10)
Time to Reach Familiarization (seconds)	59.67 (101.35)	46.70 (25.44)	50.02 (55.78)
Average Run Duration (seconds)	5.11 (3.43)	4.56 (2.67)	4.70 (2.88)

*N* sample size, *SD* standard deviation.

In the ECHO.CA.IL cohort, the GM of ΣAA was 0.20 μM (GSD = 2.68). Among the individual analytes, the GM was highest for aniline (0.24 ng/mL) and lowest for *o*-anisidine (0.25 ng/mL) (Table 2). Distributions of AAs were comparable across cohorts, and the only notable difference was that average levels of 4-chloroaniline and *o/m*-toluidine were higher in IKIDS compared to CIOB (e.g., GM of 4-chloroaniline was 1.37 ng/mL in IKIDS and 0.48 ng/mL in CIOB) (Table 2). We observed that the GM of aniline was higher among those who were married or living with a partner (9.58 μM) compared to those who were single (5.69 μM), and slightly higher among those who did not have a college degree (10.5 μM) compared to those with a graduate degree (9.78 μM) (Table S2). We found that ΣAA was strongly correlated with aniline (ρ = 0.78) and moderately to weakly correlated with other AAs (ρ ranging from 0.12 to 0.56) (Fig. S1).

**Table 2:** Distributions of aromatic amines (AAs) and cotinine measured in second trimester urine samples among a sample of the Chemical in Our Bodies (CIOB) cohort, Illinois Kids Developmental Study (IKIDS), and pooled ECHO.CA.IL cohort.

Selected Percentiles									
	*N*	% Above the Limit of Detection	Geometric Mean	Geometric Standard Deviation	Minimum	25^th^	50^th^	75^th^	Maximum
${{\boldsymbol{\sum }}}\,$**AA (μM)**									
CIOB	64		0.18	2.89	0.01	0.12	0.16	0.25	18.63
IKIDS	186		0.2	2.62	0	0.13	0.19	0.3	58.63
ECHO.CA.IL	250		0.2	2.68	0	0.13	0.19	0.28	58.63
**Aniline (ng/mL)**									
CIOB	64	96%	10.68	2.82	0.07	7.16	11.6	16.16	792
IKIDS	186	96%	8.79	3.41	0.07	7.42	10.44	15	113
ECHO.CA.IL	250		9.24	3.26	0.07	7.24	10.7	15.28	792
**Cotinine (ng/mL)**									
CIOB	64	53%	0.54	9.68	0.07	0.07	0.68	2.46	3240
IKIDS	186	55%	0.71	8.68	0.07	0.07	1.36	3.69	10300
ECHO.CA.IL	250		0.66	8.92	0.07	0.07	1.12	3.22	10300
***o/m*-toluidine (ng/mL)**									
CIOB	64	88%	0.81	4.78	0.04	0.48	1.12	2.67	6.66
IKIDS	186	93%	1.65	4.43	0.04	0.94	1.78	3.82	97.6
ECHO.CA.IL	250		1.37	4.64	0.04	0.78	1.55	3.4	97.6
**4-chloroaniline (ng/mL)**									
CIOB	64	86%	0.48	3.99	0.04	0.28	0.64	1.14	7.7
IKIDS	186	89%	1.37	5.38	0.04	0.64	1.1	2.95	192
ECHO.CA.IL	250		1.05	5.32	0.04	0.53	1	2.27	192
***o*-anisidine (ng/mL)**									
CIOB	64	68%	0.18	6.98	0.02	0.02	0.18	1.03	3.88
IKIDS	186	67%	0.28	5.32	0.03	0.03	0.46	0.94	9.89
ECHO.CA.IL	250		0.25	5.76	0.02	0.03	0.41	0.97	9.89
**4,4’methylenedianiline (ng/mL)**									
CIOB	64	83%	0.71	4.74	0.02	0.51	0.92	1.54	36.6
IKIDS	186	84%	0.63	6.15	0.03	0.38	1.17	2.06	34.2
ECHO.CA.IL	250		0.65	5.76	0.02	0.48	1.12	2	36.6

*N* sample size, *SD* standard deviation. LOD for aniline is 0.1 ng/mL, cotinine is 0.1 ng/mL, *o/m*-toluidine is 0.05 ng/mL, 4-chloraniline is 0.5 ng/mL, o-anisidine is 0.03 ng/mL, and 4,4’methylenedianiline is 0.03 ng/mL.

**Fig. 1:**
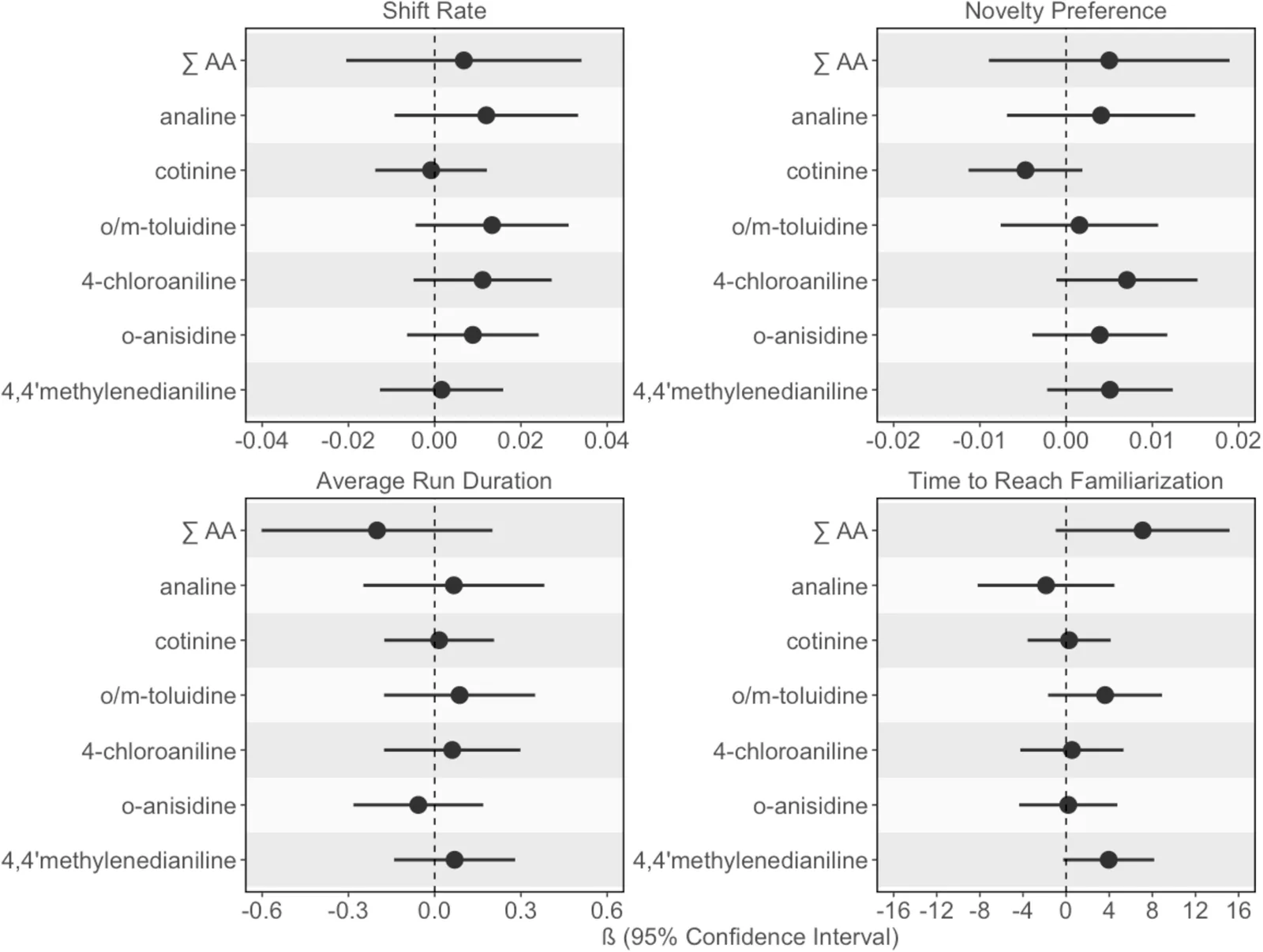
Associations between natural log transformed aromatic amines (AAs) and cotinine measured in second trimester urine with visual recognition memory outcomes assessed at 7.5 months within a subset of the ECHO.CA.IL cohort (shift rate, novelty preference, average run duration, time to reach familiarization), estimated using adjusted linear regression models (*N* = 233). Note: Models are adjusted for urinary specific gravity, maternal age, maternal education, pre-pregnancy body mass index, child age at VRM assessment, child sex, and cohort.

We observed that natural log unit increases in ΣAA, o/m-toluidine, and 4,4’methylenediailiine were individually associated with an increase in time to reach familiarization (β = 7.1, 95% confidence interval [CI] = -0.97, 15.16; β = 3.61, 95% CI = -1.68, 8.9; β = 3.95, 95% CI = -0.27, 8.17, respectively) (Fig. 1, Table S3). Most AAs were associated with an increase in shift rate (e.g., β = 0.01, 95% CI = 0.0, 0.03 for *o/m*-toluidine and 4-chloroaniline). Similarly, 4-chloroaniline, *o*-anisidine, and 4,4’methylenedianiline were associated with modest increases in novelty preference. In contrast, a natural log increase in cotinine was associated with reductions in novelty preference (β=-0.005, 95% CI = -0.011, 0.00). No notable associations were observed with average run duration (Fig. 1, Table S3). Associations were similar in cohort-specific models, although CIs were wider than what was observed for pooled models, likely reflecting smaller sample sizes across individual cohorts (Table S3). Associations were also consistent in models minimally adjusted for urinary specific gravity (Table S4).

**Fig. 2:**
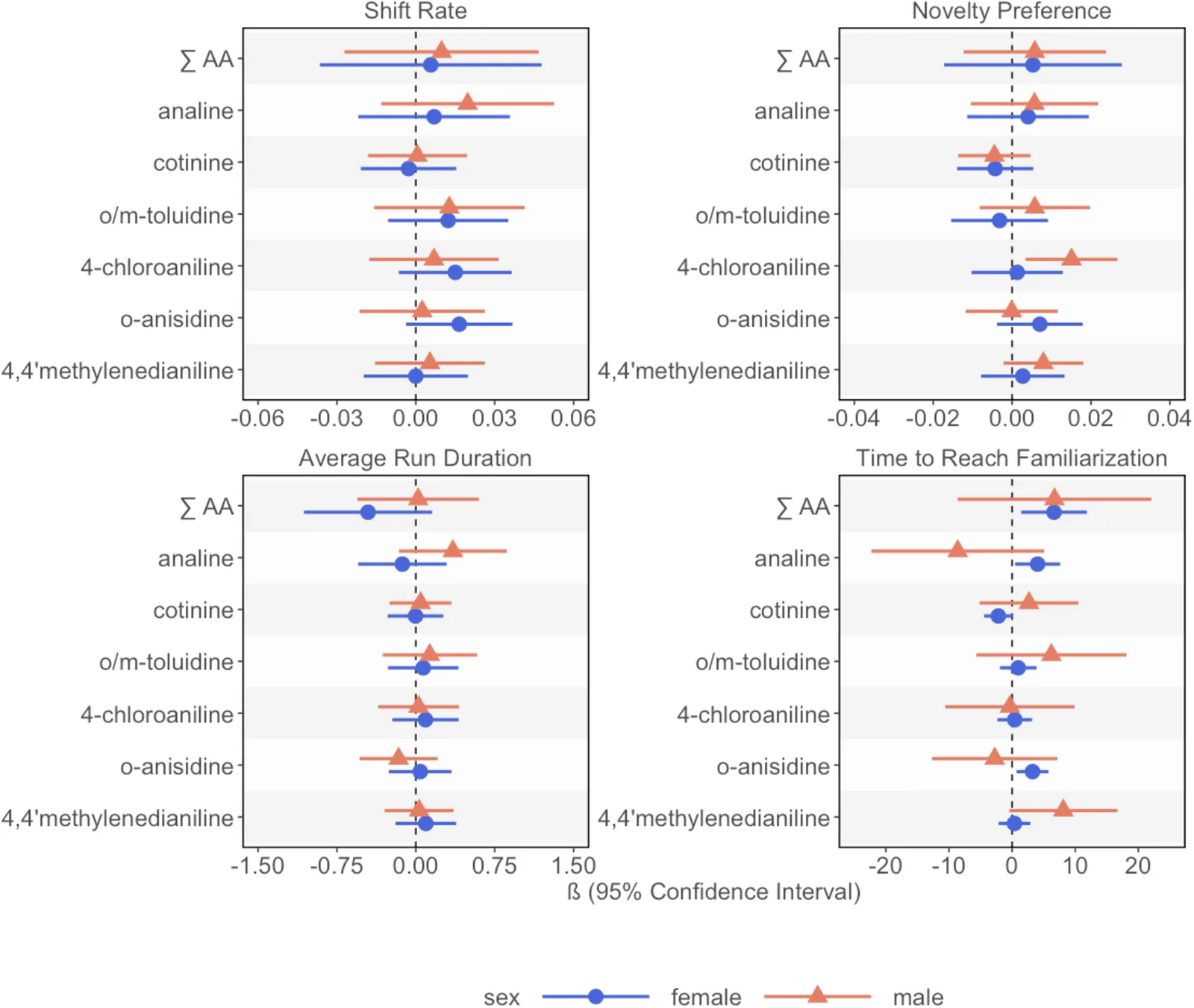
Associations between natural log transformed aromatic amines (AAs) and cotinine measured in second trimester urine with visual recognition memory outcomes assessed at 7.5 months within a subset of the ECHO.CA.IL cohort (shift rate, novelty preference, average run duration, time to reach familiarization), estimated using adjusted linear regression models stratified by child sex (*N* = 125 for females and *N* = 108 for males). Note: Models are adjusted for urinary specific gravity, maternal age, maternal education, pre-pregnancy body mass index, child age at VRM assessment, and cohort.

When examining effect modification by infant sex, we observed that aniline was associated with an increase in average run duration only among males (β_male_ = 0.35, 95% CI = -0.16, 0.86; β_female_ = -0.13, 95% CI = -0.55, 0.29, p-interaction=0.20). Similarly, 4-chloroaniline was positively associated with novelty preference only among males (Fig. 2, Table S5). In contrast, aniline and *o*-anisidine were associated with increases in time to reach familiarization only among females (e.g., for aniline, β_female_ = 4.03, 95% CI = 0.55, 7.62, β_male_ = -8.61, 95% CI = -22.29, 5.07, p-interaction = 0.06) (Fig. 2, Table S5). In models restricted to IKIDS which examined effect modification by set, we observed evidence of significant interaction between 4,4’methylenedianiline and set when novelty preference was the outcome; cotinine and set when time to reach familiarization and novelty preference were the outcomes; and *o*-anisidine and set when time to reach familiarization was the outcome (all p-interactions<0.10) (Table S6). Within IKIDS, we also examined 3-way interactions for sex, set, and AAs, but observed no evidence of 3-way interactions (data not shown).

When examining the univariate exposure-response functions obtained from BKMR (Figs. S2–S5), we observed that associations between aniline, cotinine, o/m-toluidine, and 4-chloroaniline with average run duration were non-linear with a downward u-shaped curve. All single variable risk summaries indicated non-significance with minimal evidence of interaction (Fig. S6).

## Discussion

We examined associations between prenatal exposure to six AAs and infant cognition within a subset of the prospective ECHO.CA.IL birth cohort. We observed that increasing exposure to ΣAA, o/m-toluidine, and 4,4’methylenediailiine was associated with increasing time to reach familiarization, an indicator of poorer visual attention. Similarly, increasing exposure to cotinine was associated with a reduction in novelty preference, suggesting poorer working memory. Many of these associations were sex-specific. The findings add to the growing literature that these high priority chemicals are developmental toxicants.

Our study is one of the very few that have examined perinatal and early life health effects associated with AA exposure in humans. We observed that ortho-anisidine was significantly associated with worsening visual attention (as indicated by increased time to reach familiarization). Notably, ortho-anisidine was not correlated with cotinine (*ρ* = 0.06), indicating that association between ortho-anisidine and time to reach familiarization is not an artifact of cotinine exposure.

In addition to contributing new insights into associations with neurodevelopment, our findings contribute to the small body of literature examining health effects of AAs exposures during pregnancy. Many adverse pregnancy outcomes may be on the causal pathway linking AA exposure to neurodevelopment. Prior work within the CIOB cohort found that *o*-anisidine was associated with increased odds of gestational diabetes among pregnant women [37]. However, effects were only observed among those carrying a male fetus [37]. Within the larger ECHO-wide cohort, numerous AAs were associated with small changes in gestational age, with directions of effect differing across analytes [31].

Aniline, the simplest AA in terms of chemical structure, was detected in the highest proportion of our study population. While aniline has well-documented commercial uses (e.g., in synthetic dyes, pigments, and polyurethane foams), it is also an end-stage metabolite of several AAs that undergo N-hydroxylation (including 4-chloroaniline and 4,4’methylenedianiline). These high concentrations may be reflective of this process [38]. Levels of aniline observed in our study are comparable to those within the larger ECHO-wide cohort [31]. indicating that exposure levels in this study are representative of exposures experienced by pregnant women in the US more broadly. Given the ubiquity of AAs at the population level, there is a critical need for future studies to identify the full scope of potential neurotoxic effects associated with these exposures.

Our findings contribute to a growing body of literature highlighting that the VRM task is sensitive to prenatal exposures. Prior research conducted in IKIDS found that prenatal exposure to several phthalates and the phenolic compound benzophenone-3 are associated with alternations in average run duration, indicating slower information processing at 7.5 months [34, 39]. Similarly, our previous work in IKIDS indicates that maternal experiences of perceived stress and stressful life events, as well as prenatal exposure to triclosan, are associated with increasing time to reach familiarization [40]. These patterns of association differ by exposure, suggesting some specificity of which underlying domains (i.e., attention, processing speed or memory) are affected. However, our prior work also found no association between prenatal PFAS exposure and the VRM task, which suggests that there appears to be some specificity in terms of what chemicals impact outcomes on this task [41]. Nonetheless, our findings presented here support that the VRM is sensitive to a certain prenatal exposures and can detect small effects in early life.

An important strength of our study is that infant cognition was assessed using an infrared eye tracker. This method reduces outcome misclassification and is an important advancement over prior studies which rely on parent self-reported measures of neurodevelopment. Further, we assessed infant cognition at 7.5 months, a timepoint that makes early interventions possible. Prior work using a non-automated VRM task has found that outcome measures included in our study are measures of memory, attention, and processing speed that are predictive of cognitive function later in development [26–28]. An additional strength of our study was that we pooled data from two prospective birth cohorts from different regions of the US, which enhances generalizability of our findings. However, most participants in these cohorts have high levels of educational attainment and the socioeconomic makeup of these study populations may limit comparability to other groups. Our assessment of AAs relied on a single urine sample obtained during mid-pregnancy. The half-life of the AAs measured in this study is between 20 and 60 hours [42] and a single urine sample may not be reflective of exposures across gestation and may have resulted in exposure misclassification. Despite this, prior work examining longitudinal AA exposure patterns over a five week period found that intraclass correlation coefficients for aniline and ortho-anisidine were 0.6 for both analytes, indicating moderate reliability [42]. Future work that quantifies AAs repeatedly over pregnancy is warranted to determine potential critical windows of susceptibility and may also help to assess whether these exposures tend to be continual across pregnancy.

To summarize, we conducted the first study to date examining associations between prenatal AAs exposure and infant cognition. We found that ΣAAs, as well as several individual analytes were associated with longer time to reach familiarization, reflecting poorer attention. Cotinine was similarly associated with reductions in novelty preference, reflecting poorer recognition memory. Future studies, including in vitro, in vivo and longitudinal assessment of AAs would help clarify the findings observed here.

## Supplementary information

Supplemental material

## Data Availability

Select de-identified data from the ECHO Program are available through NICHD’s Data and Specimen Hub (DASH). Information on study data not available on DASH, such as some Indigenous datasets, can be found on the ECHO study DASH webpage.
