# Supplementary material for: Prenatal exposure to aromatic amines and associations with infant cognition in the ECHO.CA.IL cohorts
Source: J Expo Sci Environ Epidemiol. 2026 Sep 21;36(6):1213–20. doi: 10.1038/s41370-026-00905-3 (PMC13727684; doi:10.1038/s41370-026-00905-3)
Supplement: Supplementary file 1 [file 41370_2026_905_MOESM1_ESM.docx]

**Supplemental Material:** Prenatal exposure to aromatic amines and associations with infant cognition in the ECHO.CA.IL cohorts

**Authors:** Stephanie M Eick^1^, Kaegan Ortlund^1^, Amy M Padula^2^, Kurunthachalam Kannan^3,4^, Andrea Aguiar^5,6^, Joshua F. Robinson,^2,7^ Rachel Morello-Frosch^2,8^, Susan L Schantz^5,6^ and Tracey J Woodruff^2^

**Affiliations:**

^1^ Gangarosa Department of Environmental Health, Rollins School of Public Health, Emory University, Atlanta, GA, USA  

^2^ Program on Reproductive Health and the Environment, Department of Obstetrics, Gynecology and Reproductive Sciences, University of California, San Francisco, San Francisco, CA, USA

^3^ Wadsworth Center, New York State Department of Health, Empire State Plaza, Albany, NY, USA

^4^ Department of Environmental Health Sciences, State University of New York at Albany, Albany, NY, USA

^5^ Beckman Institute for Advanced Science and Technology, University of Illinois Urbana-Champaign, Champaign, Illinois, USA

^6^ Department of Comparative Biosciences, University of Illinois Urbana-Champaign, Champaign, Illinois, USA

^7^ Center for Reproductive Sciences, Department of Obstetrics, Gynecology and Reproductive Sciences, University of California, San Francisco, San Francisco, CA, USA

^8^ Department of Environmental Science, Policy and Management and School of Public Health, University of California, Berkeley, Berkeley, CA, USA.

Figure S1: Spearman correlation matrix reflecting correlations between natural log transformed aromatic amines (AAs) measured in second trimester urine within a subset of the ECHO.CA.IL cohort (N=250).

Table S1: Distribution of demographic characteristics & visual recognition memory (VRM) outcomes in the larger Chemical in Our Bodies cohort (CIOB), Illinois Kids Developmental Study cohort (IKIDS), and pooled ECHO.CA.IL cohort.

|  | **CIOB** | **IKIDS** | **ECHO.CA.IL** |
| --- | --- | --- | --- |
|  | **N = 892** | **N = 563** | **N = 1455** |
|  | **N (%) or**  **Mean (SD)** | **N (%) or**  **Mean (SD)** | **N (%) or**  **Mean (SD)** |
| **Demographics** |  |  |  |
| Maternal Age (years) | 32.28 (5.35) | 30.29 (4.19) | 31.51 (5.03) |
| Missing | 1 |  | 1 |
| Pre-pregnancy body mass index (kg/m^2^) | 25.67 (5.69) | 26.53 (6.73) | 26.04 (6.17) |
| Missing | 148 | 4 | 152 |
| Marital Status |  |  |  |
| Married/Living with a partner | 710 (90%) | 492 (87%) | 1,202 (89%) |
| Single | 83 (10%) | 71 (13%) | 154 (11%) |
| Missing | 99 |  | 99 |
| Maternal Education |  |  |  |
| <College degree | 302 (36%) | 111 (20%) | 413 (29%) |
| College degree | 215 (26%) | 197 (35%) | 412 (29%) |
| Graduate degree | 324 (39%) | 255 (45%) | 579 (41%) |
| Missing | 51 |  | 51 |
| Maternal Race/Ethnicity |  |  |  |
| White | 374 (42%) | 451 (80%) | 825 (57%) |
| Black/Multi-racial | 94 (11%) | 66 (12%) | 160 (11%) |
| Asian/Native Hawaiian/Pacific Islander/American Indian/ Alaska Native | 160 (18%) | 31 (5.5%) | 191 (13%) |
| Hispanic | 253 (29%) | 15 (2.7%) | 268 (19%) |
| Missing | 11 |  | 11 |
| Parity |  |  |  |
| No prior births | 444 (50%) | 225 (40%) | 669 (46%) |
| 1 or more prior births | 439 (50%) | 338 (60%) | 777 (54%) |
| Missing | 9 |  | 9 |

Abbreviations: N, sample size; SD, standard deviation.

Table S2: Geometric mean (GM) and geometric standard deviation (GSD) of natural log transformed aromatic amines (AAs) and cotinine measured in second trimester urine across strata of selected demographic characteristics in a subset of the ECHO.CA.IL cohort.

|  | **∑ AA (μM)** | | **aniline (ng/mL)** | **4-chloroaniline (ng/mL)** | **cotinine (ng/mL)** | **4,4'methylenedianiline (n/gmL)** | ***o*-anisidine (ng/mL)** | ***o/m*-toluidine (ng/mL)** |
| --- | --- | --- | --- | --- | --- | --- | --- | --- |
|  | **GM (GSD)** | | **GM (GSD)** | **GM (GSD)** | **GM (GSD)** | **GM (GSD)** | **GM (GSD)** | **GM (GSD)** |
| **Marital Status** | |  |  |  |  |  |  |  |
| Married/Living with a partner | 0.2 (2.74) | | 9.58 (3.11) | 1.09 (5.36) | 0.58 (8.73) | 0.66 (5.71) | 0.25 (5.73) | 1.41 (4.55) |
| Single | 0.17 (1.96) | | 5.69 (5.46) | 0.68 (4.86) | 3.51 (6.34) | 0.58 (7.05) | 0.18 (6.53) | 0.94 (6.27) |
| **Maternal Education** | |  |  |  |  |  |  |  |
| <College degree | 0.29 (3.5) | | 10.48 (1.67) | 1.44 (4.1) | 4.87 (10.69) | 1.09 (5.43) | 0.23 (5.98) | 1.67 (2.73) |
| College degree | 0.17 (2.5) | | 8.03 (3.8) | 0.93 (4.68) | 0.47 (7.51) | 0.56 (5.86) | 0.21 (6.02) | 1.34 (5.4) |
| Graduate degree | 0.19 (2.5) | | 9.78 (3.37) | 1.03 (6.19) | 0.45 (6.72) | 0.62 (5.7) | 0.28 (5.53) | 1.32 (4.8) |
| **Maternal Race/Ethnicity** | |  |  |  |  |  |  |  |
| White | 0.2 (2.77) | | 9.29 (3.37) | 1.09 (5.89) | 0.54 (8.43) | 0.69 (5.31) | 0.25 (5.75) | 1.47 (4.9) |
| Black/Multi-racial | 0.23 (2.88) | | 10.15 (3.11) | 1.04 (4.13) | 1.61 (14.82) | 0.53 (6.21) | 0.2 (6.02) | 1.24 (3.76) |
| Asian/Native Hawaiian/Pacific Islander/American Indian/ Alaska Native | 0.16 (2.14) | | 8.88 (2.14) | 0.87 (3.77) | 0.77 (5.89) | 0.53 (7.42) | 0.31 (6.13) | 1.05 (3.67) |
| Hispanic | 0.19 (2.23) | | 7.53 (5.04) | 0.89 (3.96) | 1.58 (8.55) | 0.65 (10.16) | 0.17 (5.1) | 1.07 (5.49) |
| **Parity** | |  |  |  |  |  |  |  |
| No prior births | 0.2 (2.51) | | 9.44 (3.05) | 1.12 (4.46) | 0.55 (9.1) | 0.81 (5.48) | 0.26 (6.12) | 1.5 (4.66) |
| 1 or more prior births | 0.19 (2.93) | | 8.98 (3.56) | 0.97 (6.54) | 0.84 (8.67) | 0.5 (6.01) | 0.24 (5.32) | 1.28 (4.48) |

Table S3: Associations between natural log transformed aromatic amines (AAs) and cotinine measured in second trimester urine with visual recognition memory outcomes assessed at 7.5 months within a subset of the Chemical in Our Bodies (CIOB) cohort, Illinois Kids Developmental Study (IKIDS), and pooled ECHO.CA.IL cohort., estimated using adjusted linear regression models (N=184 for IKIDS, N=49 for CIOB, and N=233).

Note: Models are adjusted for urinary specific gravity, maternal age, maternal education, pre-pregnancy body mass index, child age at VRM assessment, and child sex. Models for IKIDS additionally adjust for set. Models for ECHO.CA.IL additionally adjust for cohort.

|  |  | **Shift Rate** | **Novelty Preference** | **Time to Reach Familiarization** | **Average Run Duration** |
| --- | --- | --- | --- | --- | --- |
|  |  | **ß (95% CI)** | **ß (95% CI)** | **ß (95% CI)** | **ß (95% CI)** |
|  | **∑ AA** | | | | |
|  | CIOB | -0.02 (-0.08, 0.04) | 0.02 (-0.01, 0.04) | 28.53 (-11.19, 68.26) | -0.22 (-1.38, 0.94) |
|  | IKIDS | 0.01 (-0.02, 0.04) | -0.01 (-0.02, 0.01) | 2.98 (-1.09, 7.04) | -0.16 (-0.59, 0.28) |
| ECHO.CA.IL | | 0.01 (-0.02, 0.03) | 0 (-0.01, 0.02) | 7.1 (-0.97, 15.16) | -0.2 (-0.6, 0.2) |
|  | **Aniline** | | | | |
|  | CIOB | 0.01 (-0.04, 0.05) | 0.02 (-0.01, 0.04) | -9.33 (-43.5, 24.84) | -0.42 (-1.39, 0.56) |
|  | IKIDS | 0.01 (-0.01, 0.04) | 0 (-0.01, 0.01) | -0.28 (-3.45, 2.88) | 0.22 (-0.12, 0.56) |
|  | ECHO.CA.IL | 0.01 (-0.01, 0.03) | 0 (-0.01, 0.01) | -1.86 (-8.21, 4.49) | 0.07 (-0.25, 0.38) |
|  | **Cotinine (continuous)** | | | | |
|  | CIOB | 0 (-0.04, 0.03) | 0.01 (0, 0.03) | 11.59 (-11.88, 35.07) | 0.58 (-0.07, 1.24) |
|  | IKDIS | 0 (-0.02, 0.01) | -0.01 (-0.02, 0) | -0.93 (-2.81, 0.95) | -0.07 (-0.27, 0.13) |
|  | ECHO.CA.IL | 0 (-0.01, 0.01) | 0 (-0.01, 0) | 0.28 (-3.58, 4.13) | 0.02 (-0.18, 0.21) |
|  | **Cotinine (binary detect versus non-detect)** | | | | |
|  | CIOB | -0.06 (-0.17, 0.05) | -0.06 (-0.11, 0) | 7.11 (-73.28, 87.5) | -2.88 (-4.99, -0.77) |
|  | IKDIS | -0.01 (-0.07, 0.05) | 0.03 (0, 0.05) | 7.87 (0.14, 15.6) | 0.1 (-0.75, 0.95) |
|  | ECHO.CA.IL | -0.01 (-0.07, 0.04) | 0.01 (-0.02, 0.04) | 8.69 (-7.13, 24.52) | -0.45 (-1.23, 0.33) |
|  | ***o/m*-toluidine** | | | | |
|  | CIOB | 0 (-0.04, 0.04) | 0 (-0.02, 0.02) | 18.42 (-10.61, 47.44) | 0.89 (0.09, 1.68) |
|  | IKDIS | 0.02 (0, 0.04) | 0 (-0.01, 0.01) | 0.35 (-2.25, 2.95) | -0.03 (-0.31, 0.25) |
|  | ECHO.CA.IL | 0.01 (0, 0.03) | 0 (-0.01, 0.01) | 3.61 (-1.68, 8.9) | 0.09 (-0.18, 0.35) |
|  | **4-chloroaniline** | | | | |
|  | CIOB | 0.03 (-0.01, 0.07) | -0.01 (-0.03, 0.01) | 0.18 (-28.95, 29.3) | 0.3 (-0.53, 1.13) |
|  | IKIDS | 0.01 (-0.01, 0.03) | 0.01 (0, 0.02) | 0.17 (-2.13, 2.47) | 0.04 (-0.21, 0.29) |
|  | ECHO.CA.IL | 0.01 (0, 0.03) | 0.01 (0, 0.02) | 0.53 (-4.26, 5.33) | 0.06 (-0.18, 0.3) |
|  | ***o*-anisidine** | | | | |
|  | CIOB | 0.01 (-0.02, 0.04) | -0.01 (-0.02, 0.01) | -4 (-25.05, 17.05) | 0.19 (-0.41, 0.79) |
|  | IKIDS | 0.01 (-0.01, 0.03) | 0.01 (0, 0.02) | 2.2 (-0.12, 4.53) | -0.11 (-0.36, 0.14) |
|  | ECHO.CA.IL | 0.01 (-0.01, 0.02) | 0 (0, 0.01) | 0.2 (-4.36, 4.76) | -0.06 (-0.28, 0.17) |
|  | **4,4'methylenedianiline** | |  |  |  |
|  | CIOB | -0.04 (-0.07, -0.01) | 0.01 (-0.01, 0.03) | 32.39 (11.11, 53.67) | 0.47 (-0.19, 1.13) |
|  | IKIDS | 0.01 (-0.01, 0.02) | 0 (0, 0.01) | -0.29 (-2.35, 1.76) | 0.01 (-0.21, 0.23) |
|  | ECHO.CA.IL | 0 (-0.01, 0.02) | 0.01 (0, 0.01) | 3.95 (-0.27, 8.17) | 0.07 (-0.14, 0.28) |

Table S4: Associations between natural log transformed aromatic amines (AAs) and cotinine measured in second trimester urine with visual recognition memory outcomes assessed at 7.5 months within a subset of the Chemical in Our Bodies (CIOB) cohort, Illinois Kids Developmental Study (IKIDS), and pooled ECHO.CA.IL cohort., estimated using linear regression models minimally adjusted for specific gravity (N=184 for IKIDS, N=49 for CIOB, and N=233).

|  |  | **Shift Rate** | **Novelty Preference** | **Time to Reach Familiarization** | **Average Run Duration** |
| --- | --- | --- | --- | --- | --- |
|  |  | **ß (95% CI)** | **ß (95% CI)** | **ß (95% CI)** | **ß (95% CI)** |
|  | **∑ AA** | | | | |
|  | CIOB | 0 (-0.05, 0.04) | 0 (-0.03, 0.03) | 17.81 (-7.9, 43.52) | -0.16 (-1.03, 0.71) |
|  | IKIDS | 0.01 (-0.02, 0.04) | 0 (-0.02, 0.01) | 2.92 (-1.06, 6.9) | -0.12 (-0.53, 0.3) |
| ECHO.CA.IL | | 0.01 (-0.02, 0.03) | 0 (-0.02, 0.01) | 7.06 (-0.3, 14.42) | -0.13 (-0.51, 0.25) |
|  | **Aniline** | | | | |
|  | CIOB | 0 (-0.05, 0.04) | 0.01 (-0.01, 0.04) | -8.31 (-33.91, 17.28) | -0.35 (-1.2, 0.5) |
|  | IKIDS | 0.01 (-0.01, 0.04) | 0 (-0.01, 0.01) | 0.18 (-2.92, 3.27) | 0.2 (-0.12, 0.52) |
|  | ECHO.CA.IL | 0.01 (-0.01, 0.03) | 0 (-0.01, 0.01) | -0.85 (-6.89, 5.18) | 0.11 (-0.2, 0.42) |
|  | **Cotinine (continuous)** | | | | |
|  | CIOB | 0 (-0.02, 0.02) | 0 (-0.01, 0.01) | 8.22 (-3.65, 20.09) | 0.22 (-0.18, 0.62) |
|  | IKDIS | 0 (-0.02, 0.01) | -0.01 (-0.02, 0) | -0.39 (-2.11, 1.33) | -0.04 (-0.22, 0.14) |
|  | ECHO.CA.IL | 0 (-0.01, 0.01) | -0.01 (-0.01, 0) | 1.54 (-1.69, 4.76) | 0.02 (-0.15, 0.18) |
|  | **Cotinine (binary detect versus non-detect)** | | | | |
|  | CIOB | -0.01 (-0.09, 0.08) | -0.03 (-0.08, 0.02) | -7.45 (-58.99, 44.1) | -1.74 (-3.4, -0.08) |
|  | IKDIS | 0 (-0.06, 0.06) | 0.03 (0, 0.06) | 6.15 (-1.21, 13.51) | 0.01 (-0.77, 0.8) |
|  | ECHO.CA.IL | -0.01 (-0.06, 0.04) | 0.01 (-0.01, 0.04) | 3.39 (-10.75, 17.53) | -0.42 (-1.14, 0.3) |
|  | ***o/m*-toluidine** | | | | |
|  | CIOB | 0 (-0.04, 0.03) | 0 (-0.02, 0.02) | 16.88 (-2.75, 36.52) | 0.19 (-0.48, 0.86) |
|  | IKDIS | 0.02 (-0.01, 0.04) | 0 (-0.01, 0.01) | 0.26 (-2.37, 2.9) | -0.03 (-0.3, 0.24) |
|  | ECHO.CA.IL | 0.01 (0, 0.03) | 0 (-0.01, 0.01) | 3.09 (-1.9, 8.09) | -0.01 (-0.26, 0.25) |
|  | **4-chloroaniline** | | | | |
|  | CIOB | 0.03 (0, 0.06) | 0 (-0.02, 0.02) | 3.69 (-16.56, 23.93) | 0.26 (-0.41, 0.93) |
|  | IKIDS | 0.01 (-0.01, 0.03) | 0.01 (0, 0.02) | 0.01 (-2.29, 2.32) | 0.05 (-0.19, 0.28) |
|  | ECHO.CA.IL | 0.01 (0, 0.03) | 0.01 (0, 0.02) | -0.11 (-4.56, 4.34) | 0.06 (-0.17, 0.28) |
|  | ***o*-anisidine** | | | | |
|  | CIOB | -0.01 (-0.03, 0.02) | -0.01 (-0.02, 0) | -5.31 (-19.5, 8.88) | 0.23 (-0.24, 0.7) |
|  | IKDIS | 0.01 (-0.01, 0.02) | 0.01 (0, 0.02) | 2.31 (0.01, 4.6) | -0.1 (-0.34, 0.15) |
|  | ECHO.CA.IL | 0 (-0.01, 0.02) | 0 (0, 0.01) | -0.22 (-4.43, 4) | 0 (-0.22, 0.21) |
|  | **4,4'methylenedianiline** | | | | |
|  | CIOB | -0.02 (-0.05, 0.01) | 0 (-0.01, 0.02) | 21.08 (5.24, 36.92) | 0.02 (-0.54, 0.58) |
|  | IKIDS | 0.01 (-0.01, 0.02) | 0 (0, 0.01) | -0.17 (-2.21, 1.88) | 0 (-0.21, 0.22) |
|  | ECHO.CA.IL | 0 (-0.01, 0.02) | 0 (0, 0.01) | 4.12 (0.15, 8.09) | 0.01 (-0.2, 0.21) |

Table S5: Associations between natural log transformed aromatic amines (AAs) and cotinine measured in second trimester urine with visual recognition memory outcomes assessed at 7.5 months within a subset of the pooled ECHO.CA.IL cohort, estimated using adjusted linear regression models and stratified by child sex (N= N=125 for females and N=108 for males).

Note: Models are adjusted for urinary specific gravity, maternal age, maternal education, pre-pregnancy body mass index, child age at VRM assessment, and cohort. P-values represent the amine*sex interaction in the model that is not stratified by child sex.

|  | **Shift Rate** | | **Novelty Preference** | | **Time to Reach Familiarization** | | **Average Run Duration** | |
| --- | --- | --- | --- | --- | --- | --- | --- | --- |
|  | **ß (95% CI)** | **p-value** | **ß (95% CI)** | **p-value** | **ß (95% CI)** | **p-value** | **ß (95% CI)** | **p-value** |
| **∑ AA** | | | | | | | |  |
| female | 0.01 (-0.04, 0.05) | 0.73 | 0.01 (-0.02, 0.03) | 0.65 | 6.64 (1.43, 11.84) | 0.85 | -0.45 (-1.06, 0.16) | 0.32 |
| Male | 0.01 (-0.03, 0.05) |  | 0.01 (-0.01, 0.02) |  | 6.71 (-8.64, 22.07) |  | 0.02 (-0.56, 0.6) |  |
| **Aniline** | | | | | | | |  |
| Female | 0.01 (-0.02, 0.04) | 0.87 | 0 (-0.01, 0.02) | 0.98 | 4.03 (0.45, 7.62) | 0.06 | -0.13 (-0.55, 0.29) | 0.20 |
| Male | 0.02 (-0.01, 0.05) |  | 0.01 (-0.01, 0.02) |  | -8.61 (-22.29, 5.07) |  | 0.35 (-0.16, 0.86) |  |
| **Cotinine** | | | | | | | |  |
| Female | 0 (-0.02, 0.02) | 0.93 | 0 (-0.01, 0.01) | 0.76 | -2.18 (-4.45, 0.08) | 0.10 | 0 (-0.27, 0.26) | 0.93 |
| Male | 0 (-0.02, 0.02) |  | 0 (-0.01, 0) |  | 2.67 (-5.17, 10.51) |  | 0.05 (-0.25, 0.34) |  |
| ***o/m*-toluidie** | | | | | | | |  |
| female | 0.01 (-0.01, 0.04) | 0.51 | 0 (-0.02, 0.01) | 1.0 | 0.96 (-1.95, 3.87) | 0.69 | 0.07 (-0.26, 0.41) | 0.83 |
| male | 0.01 (-0.02, 0.04) |  | 0.01 (-0.01, 0.02) |  | 6.22 (-5.68, 18.12) |  | 0.13 (-0.32, 0.58) |  |
| **4-chloroaniline** | | | | | | | |  |
| female | 0.01 (-0.01, 0.04) | 0.20 | 0 (-0.01, 0.01) | 0.48 | 0.96 (-1.95, 3.87) | 0.56 | 0.09 (-0.22, 0.41) | 0.71 |
| male | 0.01 (-0.02, 0.03) |  | 0.02 (0, 0.03) |  | 6.22 (-5.68, 18.12) |  | 0.03 (-0.36, 0.41) |  |
| ***o*-anisidine** | | | | | | | |  |
| female | 0.02 (0, 0.04) | 0.16 | 0.01 (0, 0.02) | 0.18 | 3.23 (0.7, 5.76) | 0.14 | 0.04 (-0.26, 0.34) | 0.42 |
| male | 0 (-0.02, 0.03) |  | 0 (-0.01, 0.01) |  | -2.76 (-12.7, 7.17) |  | -0.16 (-0.54, 0.21) |  |
| **4,4'methylenedianiline** | | | | | | | |  |
| female | 0 (-0.02, 0.02) | 0.50 | 0 (-0.01, 0.01) | 0.42 | 0.37 (-2.14, 2.88) | 0.04 | 0.09 (-0.19, 0.38) | 0.68 |
| male | 0.01 (-0.02, 0.03) |  | 0.01 (0, 0.02) |  | 8.11 (-0.45, 16.68) |  | 0.03 (-0.3, 0.36) |  |

Table S6: Associations between natural log transformed aromatic amines (AAs) and cotinine measured in second trimester urine with visual recognition memory outcomes assessed at 7.5 months within a subset of the Illinois Kids Developmental Study, estimated using adjusted linear regression models and stratified by set (N=90 for set 1 and N=94 for set 2).

Note: Models are adjusted for urinary specific gravity, maternal age, maternal education, pre-pregnancy body mass index, child age at VRM assessment, and child sex. P-values represent the amine*set interaction in the model that is not stratified by set.

|  | **Shift Rate** | | **Novelty Preference** | | **Time to Reach Familiarization** | | **Average Run Duration** | |
| --- | --- | --- | --- | --- | --- | --- | --- | --- |
|  | **ß (95% CI)** | **p-value** | **ß (95% CI)** | **p-value** | **ß (95% CI)** | **p-value** | **ß (95% CI)** | **p-value** |
| **∑ AA** | | | | | | | | |
| 1 | 0.01 (-0.04, 0.05) | 0.33 | -0.01 (-0.03, 0.01) | 0.78 | 5.15 (-1.32, 11.62) | 0.22 | -0.28 (-0.91, 0.34) | 0.64 |
| 2 | 0.03 (-0.02, 0.08) |  | 0 (-0.03, 0.03) |  | -0.3 (-4.91, 4.3) |  | 0.06 (-0.61, 0.72) |  |
| **Aniline** | | | | | | | | |
| 1 | 0.00 (-0.04, 0.05) | 0.27 | -0.01 (-0.03, 0.01) | 0.96 | -0.77 (-6.93, 5.4) | 0.93 | 0.24 (-0.35, 0.83) | 0.84 |
| 2 | 0.03 (-0.01, 0.06) |  | 0 (-0.02, 0.01) |  | -0.59 (-3.63, 2.46) |  | 0.27 (-0.17, 0.71) |  |
| **Cotinine** | | | | | | | | |
| 1 | 0.00(-0.02, 0.03) | 0.87 | -0.01 (-0.02, 0) | 0.53 | 1.01 (-2.3, 4.32) | 0.02 | -0.29 (-0.61, 0.02) | 0.06 |
| 2 | 0.00 (-0.02, 0.02) |  | -0.01 (-0.02, 0) |  | -2.46 (-4.35, -0.57) |  | 0.15 (-0.13, 0.43) |  |
| ***o/m*-toluidine** | | | | | | | | |
| 1 | 0.00 (-0.03, 0.04) | 0.44 | 0 (-0.02, 0.01) | 0.56 | 1.85 (-2.93, 6.62) | 0.48 | 0.06 (-0.4, 0.52) | 0.43 |
| 2 | 0.02 (0.00, 0.05) |  | 0 (-0.01, 0.02) |  | -0.63 (-3.12, 1.86) |  | -0.11 (-0.47, 0.25) |  |
| **4-chloroaniline** | | | | | | | | |
| 1 | 0.00 (-0.02, 0.03) | 0.53 | 0 (-0.01, 0.01) | 0.72 | 0.73 (-3.48, 4.93) | 0.84 | 0.06 (-0.35, 0.46) | 0.66 |
| 2 | 0.01 (-0.01, 0.04) |  | 0.01 (0, 0.02) |  | -0.32 (-2.51, 1.88) |  | 0.01 (-0.31, 0.33) |  |
| ***o*-anisidine** | | | | | | | | |
| 1 | 0.01 (-0.01, 0.04) | 0.97 | 0 (-0.01, 0.01) | 0.46 | 5.67 (1.59, 9.74) | <0.01 | -0.30 (-0.71, 0.1) | 0.27 |
| 2 | 0.02 (-0.01, 0.04) |  | 0.01 (0, 0.02) |  | -1.18 (-3.52, 1.15) |  | 0.04 (-0.29, 0.38) |  |
| **4,4'methylenedianiline** | | | | | | | | |
| 1 | 0.01 (-0.02, 0.03) | 0.59 | 0.01 (0, 0.02) | 0.01 | -0.14 (-3.9, 3.62) | 0.89 | -0.09 (-0.45, 0.27) | 0.39 |
| 2 | 0.01 (-0.01, 0.03) |  | -0.01 (-0.02, 0) |  | -0.28 (-2.28, 1.71) |  | 0.09 (-0.2, 0.38) |  |

Figure S2: Univariate exposure-response functions and 95% credible intervals for the change in shift rate resulting from individual natural log transformed aromatic amines and cotinine, measured in second trimester urine, while holding all the remaining exposures in the mixture fixed at their median concentration, estimated using BKMR in a subset of the ECHO.CA.IL cohort (N=232).

Note: Models are adjusted for urinary specific gravity, maternal age, maternal education, pre-pregnancy body mass index, child age at VRM assessment, child sex, and cohort.

Figure S3: Univariate exposure-response functions and 95% credible intervals for the change in novelty preference resulting from individual natural log transformed aromatic amines and cotinine, measured in second trimester urine, while holding all the remaining exposures in the mixture fixed at their median concentration, estimated using BKMR in a subset of the ECHO.CA.IL cohort (N=232).

Note: Models are adjusted for urinary specific gravity, maternal age, maternal education, pre-pregnancy body mass index, child age at VRM assessment, child sex, and cohort.

Figure S4: Univariate exposure-response functions and 95% credible intervals for the change in average run duration resulting from individual natural log transformed aromatic amines and cotinine, measured in second trimester urine, while holding all the remaining exposures in the mixture fixed at their median concentration, estimated using BKMR in a subset of the ECHO.CA.IL cohort (N=233).

Note: Models are adjusted for urinary specific gravity, maternal age, maternal education, pre-pregnancy body mass index, child age at VRM assessment, child sex, and cohort.

Figure S5: Univariate exposure-response functions and 95% credible intervals for the change in time to reach familiarization resulting from individual natural log transformed aromatic amines and cotinine, measured in second trimester urine, while holding all the remaining exposures in the mixture fixed at their median concentration, estimated using BKMR in a subset of the ECHO.CA.IL cohort (N=232).

Note: Models are adjusted for urinary specific gravity, maternal age, maternal education, pre-pregnancy body mass index, child age at VRM assessment, child sex, and cohort.

Figure S6: Single variable single risk summaries reflecting the contribution of individual natural log transformed aromatic amines and cotinine, measured in second trimester measure, estimated using BKMR in a subset of the ECHO.CA.IL cohort (N=232).

Note: Models are adjusted for urinary specific gravity, maternal age, maternal education, pre-pregnancy body mass index, child age at VRM assessment, child sex, and cohort.
